# *Aplysia* Neurons as a Model of Alzheimer’s Disease: Shared Genes and Differential Expression

**DOI:** 10.1007/s12031-021-01918-3

**Published:** 2021-10-18

**Authors:** Nicholas S. Kron, Lynne A. Fieber

**Affiliations:** Department of Marine Biology and Ecology, Rosenstiel School of Marine and Atmospheric Science, University of Miami, 4600 Rickenbacker Cswy, Miami, FL 33149 USA

**Keywords:** Beta-amyloid, Tau, Neuroinflammation, Invertebrate model

## Abstract

Although Alzheimer’s disease (AD) is the most common form of dementia in the United States, development of therapeutics has proven difficult. Invertebrate alternatives to current mammalian AD models have been successfully employed to study the etiology of the molecular hallmarks of AD. The marine snail *Aplysia californica* offers a unique and underutilized system in which to study the physiological, behavioral, and molecular impacts of AD. Mapping of the *Aplysia* proteome to humans and cross-referencing with two databases of genes of interest in AD research identified 898 potential orthologs of interest in *Aplysia*. Included among these orthologs were alpha, beta and gamma secretases, amyloid-beta, and tau. Comparison of age-associated differential expression in *Aplysia* sensory neurons with that of late-onset AD in the frontal lobe identified 59 ortholog with concordant differential expression across data sets. The 21 concordantly upregulated genes suggested increased cellular stress and protein dyshomeostasis. The 47 concordantly downregulated genes included important components of diverse neuronal processes, including energy metabolism, mitochondrial homeostasis, synaptic signaling, Ca^++^ regulation, and cellular cargo transport. Compromised functions in these processes are known hallmarks of both human aging and AD, the ramifications of which are suggested to underpin cognitive declines in aging and neurodegenerative disease.

**Supplementary Information:**

The online version contains supplementary material available at 10.1007/s12031-021-01918-3.

## Introduction

Aging in humans is often accompanied by progressive declines in cognitive capabilities that can result in the inability to perform basic tasks, known clinically as dementia (Weller and Budson 2018). By far the most common of these dementias is Alzheimer’s disease (AD), accounting for up to 80% of dementia cases (Crous-Bou et al. 2017). In addition to neurodegeneration, AD is distinguished from other dementias by the presence of two types of protein aggregates, amyloid-beta (Aβ) plaques and hyperphosphorylated tau protein neurofibrillary tangles, in addition to neurodegeneration (Jack et al. 2018). As of 2014, despite more than 30 years of clinical research, only five drugs had been identified as sufficiently safe and effective for international marketing approval, and these provide mostly modest clinical effects (Schneider et al. 2014). The difficulty in studying this illness in living patients coupled with a complex etiology are major hurdles to the study of AD and development of effective drugs to treat it.

One factor that may contribute to the difficulty in AD research thus far is the inability of many model systems to recapitulate the complex nature of the disease. Medina and Avila (2014) assert that an ideal AD model should be able to integrate the genetic, environmental, and aging factors that contribute to AD disease progression. Unfortunately, many current models often address only one factor in isolation (Medina and Avila 2014). However, invertebrate models offer possible alternatives in modeling the complex states which give rise to AD (Calahorro and Ruiz-Rubio 2011; Fernandez-Funez et al. 2015; Sharma et al. 2017). Not only are these models often faster, cheaper, and in line with ethical efforts to reduce the use of vertebrates in research, but they also offer unique investigative techniques or more amenable environments for study when compared to vertebrate models (Alexander et al. 2014; Gotz and Ittner 2008; Link 2005; Moloney et al. 2010; Prussing et al. 2013; Sharma et al. 2017; Surguchov 2021).

Invertebrate models have provided an alternative approach to traditional mammalian models and have been instrumental in elucidating key components of disease progression in AD and AD-related dementias (ADRD). The tractability of behavioral phenotypes and molecular techniques in *Drosophila melanogaster* and *Caenorhabditis elegans* have made these two popular invertebrate models effective tools in investigating disease mechanisms of AD and ADRD and for drug target discovery in AD and ADRD. For example, the molecular basis for Aβ and tau aggregation and toxicity were elucidated via these model systems (Fernandez-Funez et al. 2015; Hannan et al. 2016).

An underutilized model system in which to study AD and ADRD is the marine gastropod *Aplysia californica* (*Aplysia*). Among the preeminent models for learning, *Aplysia* is a well-described neural model ideal for the integrated study of learning and behavior at the molecular, cellular, neural-circuit, and whole organism levels (Baxter and Byrne 2006; Carew et al. 1983; Castellucci et al. 1970; Cleary et al. 1998; Kindy et al. 1991; Klein et al. 1982; Kupfermann 1974; Moroz 2011; Moroz et al. 2006). Due to an annual life span and a well-mapped nervous system, *Aplysia* has also proven to be an excellent model for investigating the effects of aging on learning, cognitive function, and neuronal physiology (Bailey et al. 1983; Hallahan et al. 1992; Kempsell and Fieber 2014, 2015a, b, 2016; Papka et al. 1981; Peretz et al. 1984; Rattan and Peretz 1981; Srivatsan and Peretz 1996). Molecular studies of the effects of aging on the transcriptomes of sensory neurons (SN) revealed similar aging signatures as those of other animals, including metabolic, proteostatic, and neuro-synaptic impairments similar to those that also occur in AD and ADRD (Greer et al. 2019; Greer et al. 2018; Kron et al. 2020). Furthermore, transcriptomic profiling of individually identified giant neurons in *Aplysia* have allowed for the investigation of the effects of aging on specific neurons (Kadakkuzha et al. 2013; Moroz and Kohn 2010, 2013). As a powerful neural aging model, *Aplysia* offers a unique system in which to study AD and ADRD in the context of the greatest risk factor for AD development.

Previously, cultured *Aplysia* neurons have been demonstrated to recapitulate AD-like taupathies when transfected with mutant human tau (Shemesh and Spira 2010). These neurons were subsequently used to investigate the efficacy of a potential AD therapeutic (Shemesh and Spira 2011). Similarly, exposure of cultured neurons from closely related *A. kurodai* to mutant human Aβ elucidated the inhibitory effects of Aβ on GABA-induced chloride currents (Sawada and Ichinose 1996). Furthermore, cultured *A. kurodai* sensory-motor neuron co-cultures were used to investigate the formation and deleterious effects of cofilin-actin rods, hypothesized to be the precursors to the protein aggregates that typify AD and ADRDs like Parkinson’s disease and amyotrophic lateral sclerosis, via overexpression of the native cofilin gene (Jang et al. 2005). Together these studies highlight the applicability of the *Aplysia* model system to allow for the study of AD in the context of behavior, genetics, and aging.

In this study, we further demonstrate that *Aplysia* offers a suitable model for the study of AD and ADRD by combing the *Aplysia* genome for potential orthologs of genes of interest in AD and ADRD. We also compare available molecular aging data of *Aplysia* sensory neurons (SN) to those of late-onset AD (LOAD) to demonstrate the capacity of *Aplysia* neurons to naturally recapitulate the preconditions and risk factors that are believed to contribute to AD development in human aging.

## Methods

### *Aplysia* Genome Annotation

The RefSeq proteome for the latest *Aplysia* genome build (AplCal3.0) was downloaded from the NCBI FTP site (https://ftp.ncbi.nlm.nih.gov/genomes/all/annotation_releases/6500/101/GCF_000002075.1_AplCal3.0/). The human UniProt proteome (UP000005640) was downloaded from the UniProt website (https://www.uniprot.org/proteomes/UP000005640) and used to construct a local blast database using the BLAST + command line tool (version 2.6.0; Camacho et al. 2009). The *Aplysia* proteome was then blasted against the human proteome, selecting only the top hit with an e value of ≤ 0.001. These *Aplysia*-to-human protein annotations were then imported into the R statistical environment and further annotated to the transcript and gene level for *Aplysia* using the latest gene feature format (gff, version 1.21) file available for AplCal3.0 at the NCBI FTP site. Human proteins were annotated to the gene level by mapping UniProt protein identifiers to human gene symbols using the *org.Hs.eg.db* R package (Carlson 2019; R Core Team 2013; Wickham et al. 2019).

### Overlap with Alzheimer’s Genes of Interest

The putative *Aplysia*-human orthologs generated in the previous section were then intersected with two genome-wide association meta-analysis-derived gene sets of Alzheimer’s-associated genes: Alzgset (Hu et al. 2017) and AlzGene (Bertram et al. 2007).

### Comparison of *Aplysia* Sensory Neuron Aging and LOAD in the Frontal Lobe

Gene sets previously identified as differentially expressed in aging in *Aplysia* SN (Greer et al. 2018; Kron et al. 2020) were collected and compared with genes identified as differentially expressed in LOAD via meta-analysis of six different frontal lobe data sets (Li et al. 2015). In their meta-analysis, Li et al. (2015) considered genes that were identified as significant and had concordant direction of expression change in at least five of the six data sets used. In our comparison with Li et al. (2015), we selected all genes marked as DE and exhibited concordant expression direction in at least two of the three *Aplysia* data sets (PVC from Greer et al. 2018, and PVC and BSC from Kron et al. 2020), and exhibited concordant expression direction in at least five human data sets from Li et al. (2015).

## Results

### *Aplysia* Proteome Annotation

Out of 26,658 unique proteins in the *Aplysia* RefSeq database, 20,495 proteins mapped to 9116 unique UniProt identifiers, equaling on average 2.3 *Aplysia* proteins per human protein. Each UniProt protein is mapped to one gene in the UP000005640 reference proteome; thus the ~ 20,500 *Aplysia* proteins were mapped to ~ 9000 human genes.

Among these putative orthologs were several human genes involved in AD and ADRD. An ortholog of amyloid precursor protein (APP) was identified in *Aplysia* previously, and here we identified two potential APP orthologs (Moroz and Kohn 2010). Similar to *Drosophila*, but unlike *C. elegans*, we identified putative *Aplysia* orthologs of both beta-secretase 1 (BACE1) and all components of the gamma-secretase complex: presenilin (PSEN), nicastrin (NCSTN), presenilin enhancer 2 (PSENEN), and two putative orthologs of anterior pharynx-defective 1 (APH1A). We also identified several potential *Aplysia* orthologs to the primary alpha secretase A disintegrin and metalloproteinase (ADAM) family members including three orthologs of ADAM10, two orthologs of ADAM12, and seven orthologs of ADAM17. Two potential orthologs of the tau protein gene MAPT were also identified.

Of interest in Parkinson’s disease, six potential orthologs of leucine-rich repeat kinase 2 (LRRK2/PARK8), along with putative orthologs of other Parkinson’s disease-associated genes such as protein deglycase DJ-1 (PARK7/DJ-1), Parkin (PRKN), Parkin coregulated gene protein (PACRG), and synphilin (SNCAIP), were identified. However, a potential ortholog for alpha-synuclein (SNCA/PARK1) was not identified.

### Overlap with Alzgset and AlzGene

**Fig. 1:**
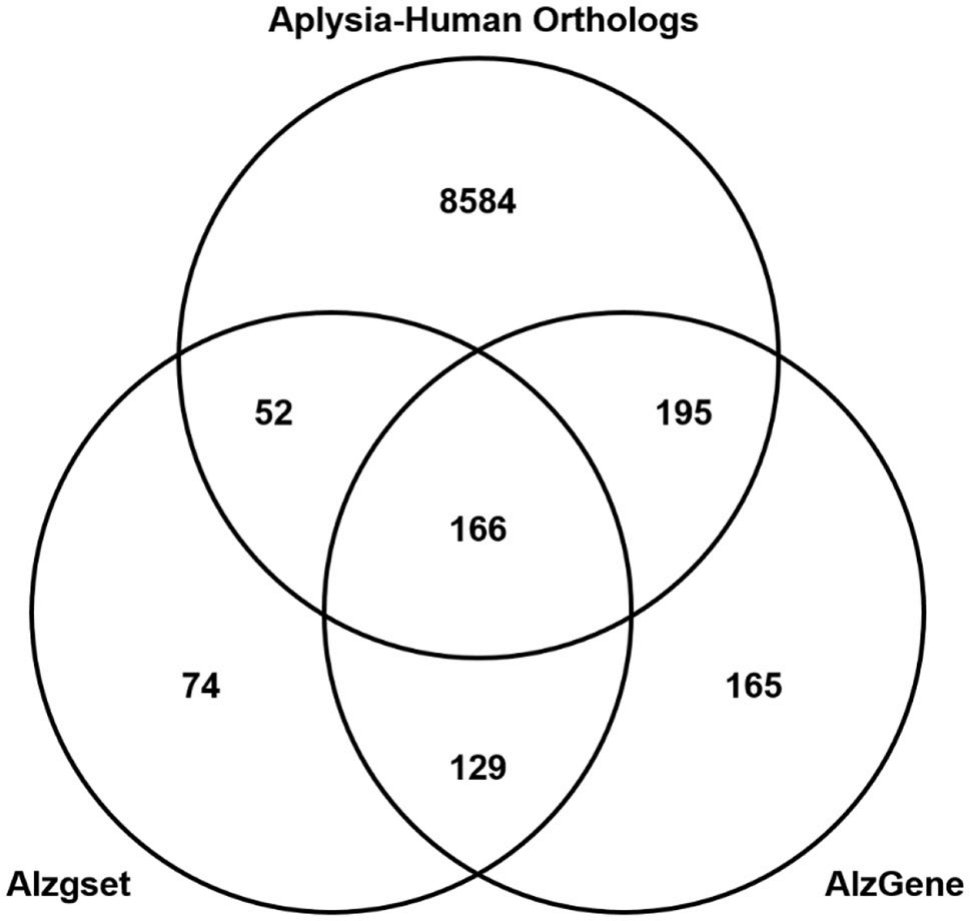
Gene set overlap of putative human orthologs found in the *Aplysia* genome with Alzheimer’s disease (AD)-associated gene databases Alzgset and AlzGene. *Aplysia* RefSeq proteins were mapped to the UniProt human protein database using the BLAST + command line tool. The two AD genes-of-interest data sets shared 295 genes. Of the more than 9000 *Aplysia*-human orthologs identified, 418 were present in either Alzgset, or AlzGene, or both. A smaller subset of 166 genes was identified as common to all three gene sets

Of the 9000 putative orthologs, 219 were present in Alzgset and 364 were present in AlzGene. Alzgset and AlzGene share 295 genes, of which 166 were among the ~ 9000 *Aplysia*-human orthologs. Considering genes from either data set, a total of 418 AD genes of interest with putative orthologs in the *Aplysia* genome were identified (Fig. 1). This corresponds to 1207 *Aplysia* transcripts from 898 *Aplysia* genes. As noted in the above section, orthologs of PSEN1, APP, and MAPT were present, along with several other Aβ- and tau-associated proteins (Table 1). The full mapping is available in Supplemental Data 1.

**Table 1:** Selection of Aβ- and tau-associated genes present in both the AlzGene and Alzgset databases that have putative *Aplysia* gene orthologs. Human gene symbols are mapped to gene name, putative *Aplysia* ortholog IDs, UniProt accession, Gene Ontology IDs, and Gene Ontology names. Genes represented were annotated for GO BP or MF associated with Aβ or tau, present in the AlzGene and Alzgset gene sets, and annotated to putative *Aplysia* gene orthologs by BLAST + with an e-value of ≤ 0.0001. Genes of high interest in AD are bolded

Gene symbol	Gene name	*Aplysia* gene	UniProt IDs	GO IDs	GO names
ADAM10	ADAM metallopeptidase domain 10	LOC101859462, LOC101851963, LOC101845373	O14672	GO:0,034,205, GO:0,042,987	Ab formation, amyloid Precursor protein catabolic process
**APH1A**	**aph-1 homolog A, gamma-secretase subunit**	**LOC101856734**	**Q96BI3**	**GO:0,034,205, GO:0,042,987, GO:0,042,982**	**Ab formation, amyloid precursor protein Catabolic process, amyloid precursor protein metabolic process**
**NCSTN**	**Nicastrin**	**LOC100533532**	**Q92542**	**GO:0,034,205, GO:0,042,987, GO:0,042,982**	**Ab formation, amyloid precursor protein catabolic process, amyloid precursor protein metabolic process**
**PSEN1**	**Presenilin 1**	**LOC100533344**	**P49768**	GO:0,034,205, GO:0,042,987, GO:0,042,982	Ab formation, amyloid precursor protein catabolic process, amyloid precursor protein metabolic process
**PSENEN**	**Presenilin enhancer, gamma-secretase subunit**	**LOC101854684**	**Q9NZ42**	**GO:0,034,205, GO:0,042,987, GO:0,042,982**	**Ab formation, amyloid precursor protein catabolic process, amyloid precursor protein metabolic process**
DYRK1A	Dual-specificity tyrosine phosphorylation-regulated kinase 1A	LOC106013836	Q13627	GO:0,034,205, GO:0,048,156	Ab formation, tau binding
ADRB2	Adrenoceptor beta 2	LOC101855541, LOC101851894, LOC101852650, LOC118478765, Apoa	P07550	GO:0,001,540	Amyloid-beta binding
APBB2	Amyloid-beta precursor protein-binding family B member 2	LOC101847028	Q92870	GO:0,001,540	Amyloid-beta binding
BCHE	Butyrylcholinesterase	LOC101862164, LOC101860246, LOC101862869, LOC101851188, LOC101856264, LOC101862414, LOC101861954, LOC101846738, LOC101862657, LOC101859867, LOC106013051, LOC101851390, LOC101854068, LOC118479136	P06276	GO:0,001,540	Amyloid-beta binding
CST3	Cystatin C	LOC101857420	P01034	GO:0,001,540	Amyloid-beta binding
EPHA4	EPH receptor A4	LOC101861456	P54764	GO:0,001,540	Amyloid-beta binding
GRIN2B	Glutamate ionotropic receptor NMDA type subunit 2B	LOC100533244	Q13224	GO:0,001,540	Amyloid-beta binding
HSPG2	Heparan sulfate proteoglycan 2	LOC101857847, LOC101859116, LOC101861971, LOC101855448, LOC101847382	P98160	GO:0,001,540	Amyloid-beta binding
LRPAP1	LDL receptor-related protein associated protein 1	LOC101847798, LOC101860965	P30533	GO:0,001,540	Amyloid-beta binding
NGFR	Nerve growth factor receptor	LOC106012918	P08138	GO:0,001,540	Amyloid-beta binding
SORL1	Sortilin-related receptor 1	LOC101857914, LOC118477251, LOC101846105	Q92673	GO:0,001,540	Amyloid-beta binding
TLR4	Toll-like receptor 4	LOC101847817, LOC101850809, LOC101860761	O00206	GO:0,001,540	Amyloid-beta binding
LDLR	Low-density lipoprotein receptor	LOC118478465	P01130	GO:0,001,540, GO:0,097,242	Amyloid-beta binding, Amyloid-beta clearance
LRP1	LDL receptor-related protein 1	LOC101849041, LOC101849281, LOC101859513, LOC100533545, LOC118478804, LOC118478805, LOC106013813, LOC106013825	Q07954	GO:0,001,540, GO:0,097,242	Amyloid-beta binding, Amyloid-beta clearance
IDE	Insulin-degrading enzyme	LOC101845820	P14735	GO:0,001,540, GO:0,097,242, GO:0,050,435	Amyloid-beta binding, Amyloid-beta clearance, Amyloid-beta metabolic process
**BACE1**	**Beta-secretase 1**	**LOC101859129**	**P56817**	**GO:0,001,540, GO:0,050,435**	A**myloid-beta binding,** A**myloid-beta metabolic process**
CHRNA7	Cholinergic receptor nicotinic alpha 7 subunit	LOC101851082, LOC101856227, LOC101862541, LOC101856484, LOC101852526, LOC101856946, LOC101852974, LOC106012547, LOC106013357, LOC101853763, LOC101845987, LOC101845835, LOC101857864, LOC101858254, LOC101860243, LOC101845238, LOC101845238, LOC101856899, LOC101856899, LOC101858495, LOC101860344, LOC101860583, LOC106012370, LOC101860114, LOC101860352, LOC101853250, LOC101853479, LOC101861149	P36544	GO:0,001,540, GO:1,904,645	Amyloid-beta binding, response to amyloid-beta
PICALM	Phosphatidylinositol-binding clathrin assembly protein	LOC101848715	Q13492	GO:0,001,540, GO:0,048,156	Amyloid-beta binding, tau binding
MME	Membrane metalloendopeptidase	LOC101861636, LOC101853869, LOC101854751	P08473	GO:0,097,242, GO:0,050,435	Amyloid-beta clearance, Amyloid-beta metabolic process
ACE	Angiotensin-converting enzyme I	LOC101850558, LOC101862115, LOC101849400, LOC101863140	P12821	GO:0,050,435	Amyloid-beta metabolic process
**APP**	**Amyloid-beta precursor protein**	**LOC118478801, LOC100533426**	**P05067**	GO:1,990,000	Amyloid fibril formation
**MAPT**	**Microtubule-associated protein tau**	**LOC101864325, LOC106010967**	**P10636**	GO:1,990,000	Amyloid fibril formation
ABCG1	ATP-binding cassette subfamily G member 1	LOC101862516	P45844	GO:0,042,987	Amyloid precursor protein catabolic process
DHCR24	24-Dehydrocholesterol reductase	LOC101864542, LOC101864542, LOC101849310	Q15392	GO:0,042,987	Amyloid precursor protein catabolic process
BIN1	Bridging integrator 1	LOC101856166	O00499	GO:0,048,156	Tau binding
CDK5	Cyclin-dependent kinase 5	LOC101853437, LOC101864023	Q00535	GO:0,048,156	Tau binding
GSK3B	Glycogen synthase kinase 3 beta	LOC100533534	P49841	GO:0,048,156	Tau binding
PIN1	Peptidyl-prolylcis/trans isomerase, NIMA-interacting 1	LOC101858155	Q13526	GO:0,048,156	Tau binding

### Comparison to LOAD Frontal Cortex Study

**Fig. 2:**
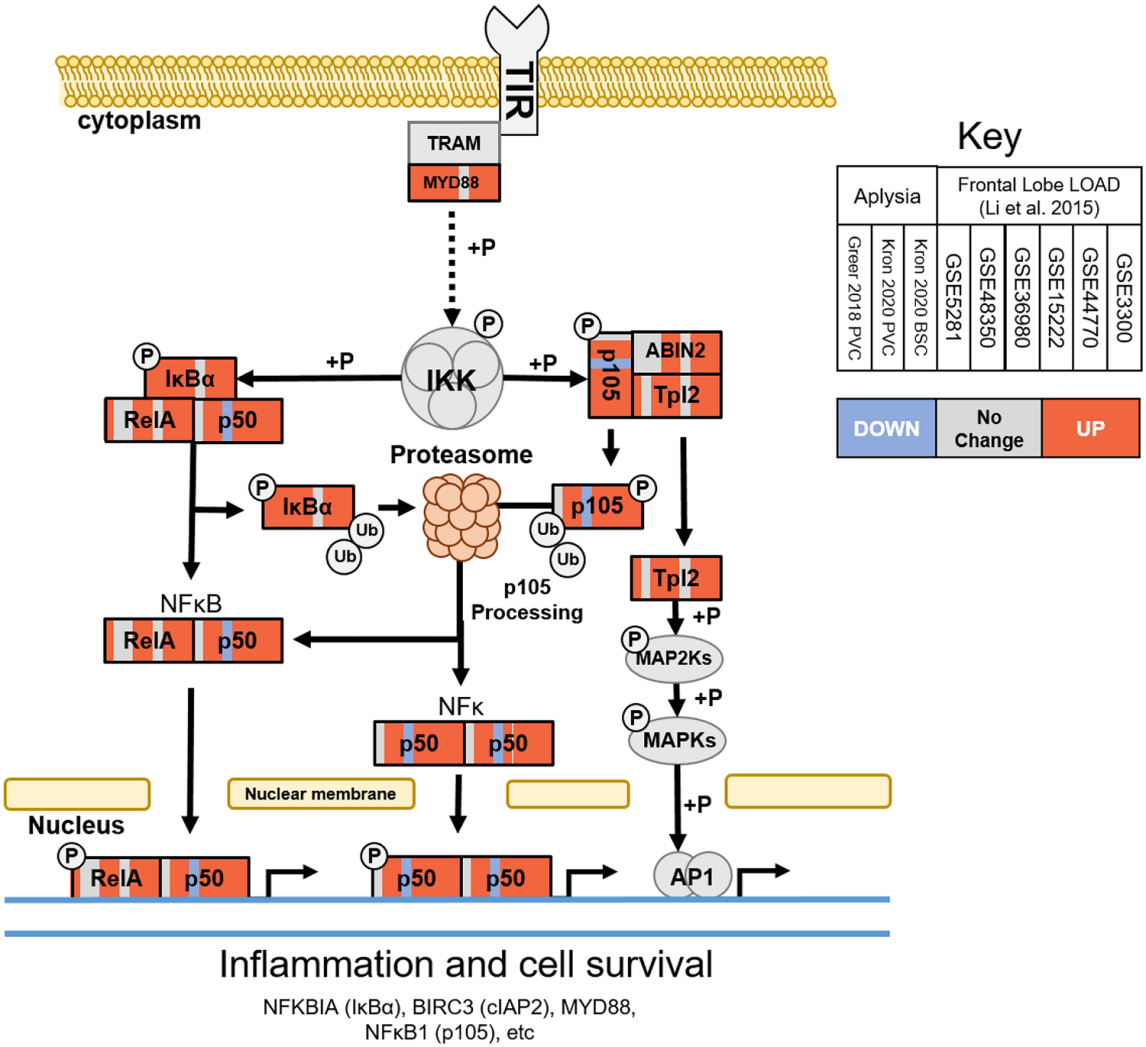
Commonly upregulated orthologs in the toll/interleukin signaling pathway between *Aplysia* SN aging and FL LOAD. Each box represents a gene and is subdivided into nine sections corresponding to analyzed data sets; the first three are *Aplysia* SN data sets and the latter six are FL LOAD results from Li et al. (2015) as demarcated in the Key. Each section is colored to indicate whether the genes were upregulated (red), downregulated (blue), or exhibited no change (gray) in the corresponding data set. Solid line arrows represent a direct interaction, while dotted lines signify indirect interaction via intermediaries. Example genes known to be upregulated by NF-kB that were common to both *Aplysia* SN aging and FL LOAD are listed at the bottom of the figure. Commonly upregulated genes include toll/interleukin signaling adapter protein MYD88 and several components of the NF-kB signaling cascade (IκΒα, NF-κΒ1, TPL2), suggesting that increased proinflammatory signaling is a common feature of *Aplysia* SN aging and FL LOAD

**Fig. 3:**
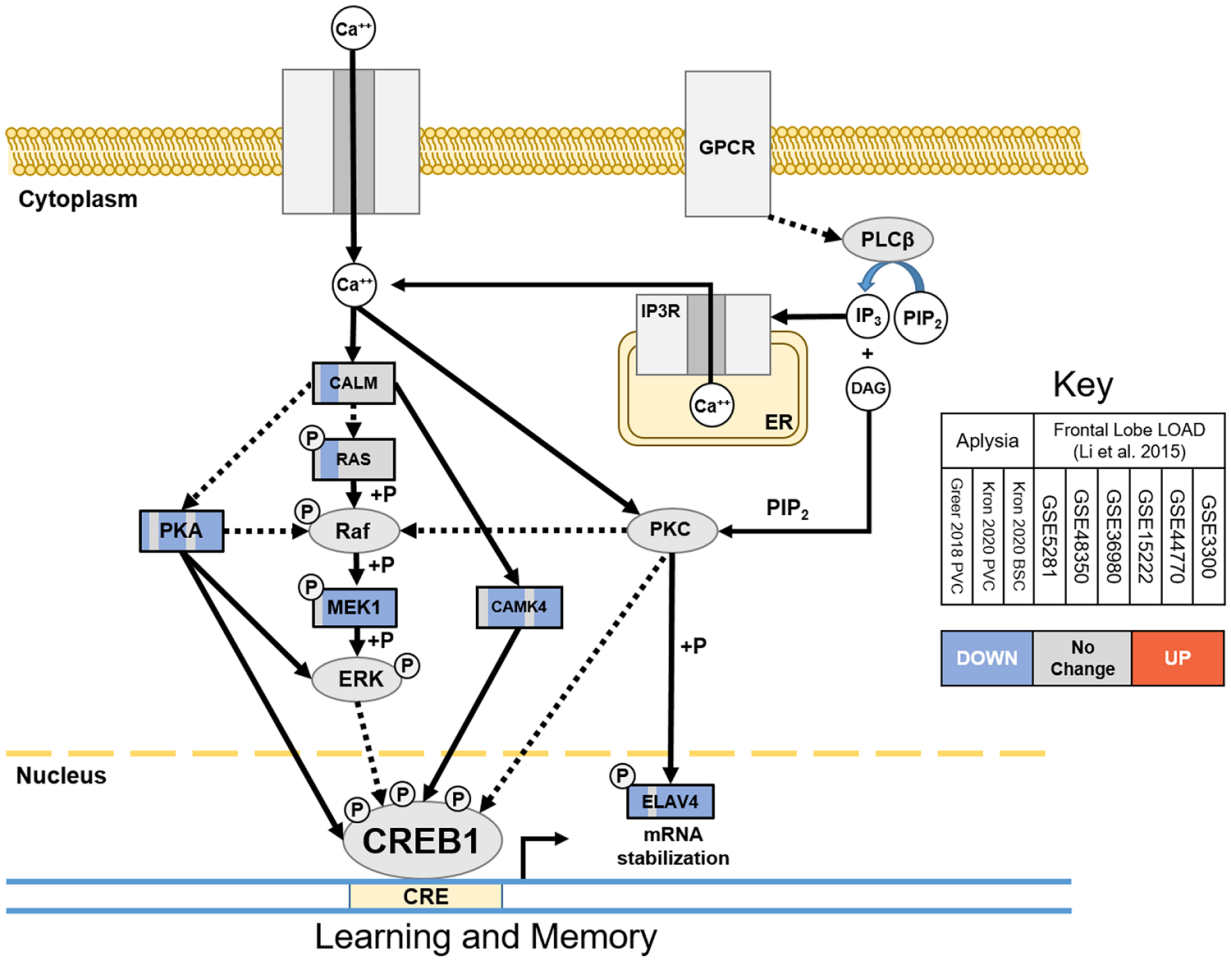
Orthologs in learning and memory pathway downregulated in common between *Aplysia* SN aging and FL LOAD. See Fig. 2 caption for diagram description. Commonly downregulated genes included major kinases of CREB1 (PKA, CAMK4, MEK1) and ELAV4, which stabilizes mRNAs of CREB1 target genes. This suggests that CREB1 signaling disruption is a common cause of cognitive impairment in *Aplysia* SN and LOAD

Comparison of differential expression in three aging *Aplysia* SN data sets with a meta-analysis of six frontal cortex LOAD (FL LOAD) data sets identified 68 putative gene orthologs concordantly differentially expressed in at least five of the FL LOAD studies and two *Aplysia* data sets. Of these genes, 21 were concordantly upregulated and 47 concordantly downregulated. Commonly upregulated genes included cellular stress-induced genes such as ANKZF1, BTG1, DDIT4L, and SSR1, as well as elements of the proinflammatory toll/interleukin receptor signaling pathways such as MYD88, NFKBIA, MAP3K8, and BIRC3 (Fig. 2 and Table 2). Commonly downregulated genes were representative of diverse processes including synaptic vesicle dynamics (SYN2, EXOC8, NAPG, SVOP, ARF3), transport of cellular cargo (DCTN6, KIFAP3, RAB6A), energy metabolism (GOT1 and 2, MDH1, CYCS, NDUFV1, PCCB), cyclic-AMP response element-binding protein (CREB)-mediated learning and memory (MAP2K1, PRKACA, CAMK4, ELAV4, Fig. 3) and mitochondrial homeostasis (GDAP1, TUSC2), among others (Table 3). The full gene list is available in Supplementary Data 2.

**Table 2:** Gene orthologs upregulated in both *Aplysia* SN aging and FL LOAD. All genes upregulated in two or more aging *Aplysia* SN differential expression data sets and five or more in meta-analysis of human frontal lobe Late Onset AD (FL LOAD) samples by Li et al. (2015). *Aplysia* RefSeq transcript identifiers, their BLAST-assigned putative human orthologs, and the e-value of the match are listed in the first three columns, with alternative names for each human gene in the fourth. The number of data sets in which these orthologs were upregulated is listed in columns 5 (*Aplysia* data sets) and 6 (Li et al. 2015 human FL LOAD data sets). Column 6 groups orthologs into broad categories relevant to aging and AD found in the discussion

*Aplysia* RefSeq Transcript	e-value	Human gene symbol	Other names	*Aplysia* data sets	FL LOAD data sets	Major category
XM_005091054	9.3E-70	ANKZ1	ANKZF1, ZNF744	3	5	Stress response (ER, ROS)
XM_013084296	5.3E-09	BIRC3	API2, MIHC, cIAP	3	6	Inflammation
XM_013088003	7.2E-12	BIRC3	API2, MIHC, cIAP	3	6	Inflammation
XM_005111747	5.3E-08	BIRC3	API2, MIHC, cIAP	2	6	Inflammation
XM_005102233	6.5E-22	BMP1	mTlD, PCP, TLD	2	5	Inflammation, cholesterol metabolism
XM_005112068	4.2E-20	BTG1	BTG1	2	6	Stress response (metabolic, ER, ROS)
XM_013080222	1.4E-86	CP3A5	CYP3A5	2	5	Lipid metabolism, cholesterol metabolism
XM_005102749	1.1E-19	DDT4L	DDIT4L, REDD2	2	6	Stress response (metabolic)
XM_013089385	5.6E-17	GA45G	GADD45G, DDIT-2, CR6	3	5	Stress response
XM_005111489	3.2E-34	IKBA	NFKBIA, MAD3, NFKBI	3	5	Inflammation
XM_013089050	4.9E-37	M3K8	MAP3K8, COT, TPL2	2	5	Inflammation
XM_005095469	0	MA2B1	MAN2B1, LAMAN, MANB	2	5	Proteostasis
NM_001204684	1.4E-135	MKNK2	MNK2, GPRK7	2	6	Inflammation
XM_005108634	2.2E-25	MLXIP	MONDOA	3	5	Energy metabolism
XM_005089580	6.6E-05	MUC1	CD227, PEM, EMA, EMA, PEMT	2	5	Stress response (ER), inflammation
XM_013081198	2.2E-15	MYD88	MYD88	3	5	Inflammation
XM_005097661	4.4E-49	NEO1	NGN, IGDCC2	2	5	Iron accumulation, inflammation
XM_005108885	4.1E-21	NFIL3	E4BP, IL3BP1	2	5	Inflammation
XM_005096173	1.7E-12	NFKB1	EBP1	2	5	Inflammation
XM_005091237	1.1E-77	SSRA	SSR1, TRAPA	2	5	Stress response (ER)
XM_005110832	7.2E-43	TISB	ZFP36L1, BRF1, ERF1, TIS11B, BERG36, RNF162B	3	6	Inflammation, cholesterol metabolism

**Table 3:** Gene orthologs downregulated in both *Aplysia* SN aging and FL LOAD. All genes downregulated in two or more aging *Aplysia* SN differential expression data sets and five or more in meta-analysis of human frontal lobe FL LOAD samples by Li et al. (2015). See Table 2 for column descriptions. A majority of shared downregulated orthologs are involved in one or more of the following processes: cellular cargo transport, endo/exocytosis, proteostasis, lipid metabolism, energy metabolism, mitochondrial homeostasis, and signaling

*Aplysia* RefSeq Transcript	e-value	Human gene symbol	Other names	*Aplysia* data sets	FL LOAD data sets	Major category
XM_005098930	0	AATM	GOT2	3	5	Energy metabolism
XM_005099066	2.5E-46	ARF3	ARF3	2	6	Cellular cargo transport
XM_005112446	2.6E-25	CISD1	ZCD1, mitoNEET	2	5	Energy metabolism
XM_013080281	3.6E-21	CNRP1	C2orf32	2	6	Signaling
XM_005098434	1.3E-59	CYC	CYCS	2	6	Energy metabolism
XM_005096347	3.9E-65	DCTN6	WS3	2	6	Cellular cargo transport
XM_005100966	1.1E-107	DECR2	PDCR, SDR17C1	2	5	Lipid metabolism
XM_005092530	2.1E-146	ELAV4	ELAVL4, HUD, PNEM	2	6	Synaptic plasticity, mRNA stabilization
XM_005112819	0	EXOC8	EXO84	2	5	Exocytosis
XM_005097581	5.5E-10	FABPH	FABP3, FABP11, MDGI	2	5	Lipid metabolism
XM_005096727	4.0E-41	GDAP1		3	5	Mitochondrial homeostasis
XM_005111161	8.8E-44	GDAP1		2	5	Mitochondrial homeostasis
NM_001204703	0	GNAO	GNAO1	3	6	Signaling, Ca^++^ homeostasis
XM_005102254	0	GNB5		2	5	Signaling, Ca^++^ homeostasis
XM_005112007	0	HMCS1	HMGCS1,HMGCS	2	5	Lipid metabolism, steroid metabolism
XM_005104774	4.8E-106	HPRT	HPRT1, HGPRT	2	6	Nucleotide salvage
XM_005102830	2.0E-07	JUPI1	ARM2, HN1	2	5	Other
NM_001204491	0	KAPCA	PRKACA, PKACA	2	5	Synaptic plasticity, Ca^++^ signaling, phosphorylation
XM_005106951	4.0E-65	KCC4	CAMK4, CAMK, CAMK-GR, CAMKIV	2	5	Synaptic plasticity, Ca^++^ signaling, phosphorylation
XM_005104905	0	KIFA3	KIFAP3, KIF3AP, SMAP	3	6	Cellular cargo transport
XM_005102605	1.4E-10	LIAT1	C17orf97	2	5	Other
XM_005098563	4.0E-171	MDHC	MDH1, MDHA	2	6	Energy metabolism
XM_005089329	0	MP2K1	MAP2K1, MEK1, PRKMK1, MKK1, MAPKK1	2	6	Synaptic plasticity, phosphorylation
XM_005098362	3.2E-56	MPND	MPND	3	5	Other
XM_005089044	7.7E-36	NDUAA	NDUFA10, CI-42kD	2	5	Energy metabolism
XM_005097418	0	NDUV1	NDUFV1, UQOR1	2	5	Energy metabolism
XM_005099251	2.6E-103	NECP1	NECAP1	2	6	Endocytosis
XM_005097828	0	ODPB	PDHB, PHE1B	3	6	Energy metabolism
XM_013084642	3.7E-89	OTUB1	OTB1, OTU1	3	6	DNA damage response
XM_013081831	0	PCCB		2	5	Lipid metabolism
XM_005089882	4.6E-28	PEX19	HK33, PXF	2	5	Lipid metabolism, proteostasis
XM_005110189	0	PFKAM	PFKM, PFKA, PFKX	2	6	Energy metabolism
XM_005109909	4.9E-74	PITH1	PITHD1, C1orf128	2	5	Transcription
XM_005097948	2.5E-50	PPAC	ACP1, LMW-PTP	2	6	Phosphorylation
XM_005097122	4.3E-133	RAB6A	RAB6	2	5	Cellular cargo transport
XM_005093164	1.7E-87	SAMC	SLC25A26	3	5	Mitochondrial homeostasis
XM_005108342	9.4E-28	SCOC	SCOCO	3	6	autophagy
XM_005093202	4.4E-78	SNAG	NAPG, SNAPG	2	6	Cellular cargo transport, endocytosis
XM_013087712	1.4E-156	SNX4		3	6	Cellular cargo transport, endocytosis, proteostasis
XM_005091494	2.5E-111	SPEE	SRM, SPS1, SRML1, SPDSY	3	5	Mitochondrial homeostasis, proteostasis
NM_001204727	5.4E-129	STAU2	STAU2	2	6	Cellular cargo transport
XM_013086901	5.0E-24	STUM	C1orf95	3	5	Other
XM_005107969	0	SVOP		2	5	Exocytosis
NM_001204483	2.7E-151	SYN2		2	5	Exocytosis
XM_013090258	1.1E-63	TTPAL	C20orf121	2	5	Other
XM_005091686	3.4E-21	TUSC2	C3orf11, FUS1, LGCC, PDAP2	2	6	Mitochondrial homeostasis, inflammation, cytokine signaling, ROS response
XM_005109532	8.7E-119	1433Z	YWHAZ, KCIP-1, 14–3-3 protein zeta/delta	2	5	Signaling, proteostasis

## Discussion

In our screening of the *Aplysia* genome for orthologs to Alzheimer’s-associated genes we identified 418 putative orthologs. Among these were orthologs of hallmark players in AD progression such as Aβ and tau.

The quintessential hallmark of AD is the formation of Aβ plaques in the nervous system. Aβ is a cleavage product of APP by the single protein beta secretase and the multi-protein gamma secretase enzymes. In contrast to beta and gamma secretases, alpha secretases process APP in a manner that does not produce Aβ. The alpha secretase ADAM10 has been demonstrated to compete with beta and gamma secretases for APP and confers protection from Aβ accumulation and tau hyperphosphorylation (Peron et al. 2018; Yuan et al. 2017). While Aβ plaques associated with AD in humans are not known to occur in invertebrates, endogenous orthologs of APP and associated secretases in *Drosophila* and *C. elegans* have been used to investigate the mechanisms by which these enzymes and cleavage byproducts function in normal and pathological conditions. This approach has shed light on the mechanisms of Aβ-related AD pathology, suggesting that *Aplysia* can be used similarly (Alexander et al. 2014; Calahorro and Ruiz-Rubio 2011; Fernandez-Funez et al. 2015; Link 2005; Prussing et al. 2013).

Neurofibrillary tangles of hyperphosphorylated tau protein are also a hallmark of AD and several ADRDs. Tau neurofibrillary tangles do not naturally occur in invertebrate models; thus previous studies of tau hyperphosphorylation using *Drosophila* and *C. elegans* expressed altered human tau in invertebrate neurons to determine its detrimental effects (Alexander et al. 2014; Calahorro and Ruiz-Rubio 2011; Fernandez-Funez et al. 2015; Hannan et al. 2016; Link 2005; Moloney et al. 2010; Prussing et al. 2013; Sharma et al. 2017). These invertebrate models have been particularly useful in screening for the effects of taupathies in the nervous system (Hannan et al. 2016). Similarly, *Aplysia* SN do not naturally form tau neurofibrillary tangles; however, expression of mutant human tau also has been performed in *Aplysia* SN, which resulted in recapitulation of AD-like taupathies (Shemesh and Spira 2010, 2011). The presence of endogenous MAPT orthologs and the demonstrated capacity to induce taupathies in cultured neurons suggest that *Aplysia* SN may also offer an effective screening tool for the effects of hallmark AD proteinopathies on neurons.

The roughly 400 other orthologs of interest in *Aplysia* offer a broad landscape for functional investigation of the effects of amyloidopathies and taupathies on individual neurons and simple neural circuits. Given the success of translating molecular mechanisms of learning and memory from *Aplysia* to higher vertebrates and humans, the potential for investigation of AD mechanisms in *Aplysia* appears promising (Abrams 2012; Bailey et al. 1983; Ezzeddine and Glanzman 2003; Glanzman 2006; Kupfermann 1974; Lin and Glanzman 1994; Martin et al. 1997; Moroz 2011). This notion is further supported by the shared differential expression of genes which are involved in processes known to play key roles in both neuronal aging and AD, including learning and memory, neuronal signaling, transport of cellular cargo, energy metabolism, proteostasis, and neuroinflammation.

Memory impairment associated with AD has been suggested to be the result of synergistic toxicity between Aβ plaques and tau neurofibrillary tangles in cognitive centers like the frontal lobe and hippocampus. Gene transcription as a result of CREB activation is essential for memory formation across *Metazoa* (Silva et al. 1998). Disruption of CREB signaling in cognitive centers has been observed in AD brains as well as rodent and neuronal models of AD and is suggested to be a major component of AD-associated cognitive impairment (Puzzo et al. 2005; Snyder et al. 2005; Tong et al. 2001; Vitolo et al. 2002; Yamamoto-Sasaki et al. 1999). Similarly, *Aplysia* SN have been demonstrated to have impaired CREB signaling in aging (Greer et al. 2018; Kempsell and Fieber 2015a). As illustrated in Fig. 3, both aged *Aplysia* SN and human FL LOAD exhibited downregulation of orthologs of CAMKIV, MAP2K1, and PRKACA. These are critical components of the Ca^++^/calmodulin (Bito et al. 1996; Hardingham et al. 1998), MEK/ERK (Grewal et al. 2000; Li et al. 2019), and PKA (Turnham and Scott 2016) signaling cascades, respectively, that activate CREB during memory formation. Furthermore, commonly downregulated ELAV4 is a key effector of PKC that plays a critical role in stabilizing the mRNA of CREB target genes, facilitating protein translation and the establishment of CREB-dependent long-term memory in both species (Anderson et al. 2001; Deschenes-Furry et al. 2006; Mirisis et al. 2021; Pascale et al. 2004). Decreased activity and expression of these genes as a result of Aβ and tau has been described previously in AD (Amadio et al. 2009; Gong et al. 2006; Hartmann et al. 2019; Vitolo et al. 2002; Yin et al. 2016b). This suggests that it is the dysregulation of key kinases and their effectors in the CREB signaling cascade that drives the cognitive impairments that typify both *Aplysia* SN aging and AD.

A mechanism by which AD is believed to impair cognitive function is via the disruption of normal vesicle dynamics and proper trafficking of cellular cargo (Barthet and Mulle 2020; Marsh and Alifragis 2018). Many of the putative orthologs downregulated in aging *Aplysia* SN and FL LOAD, namely NAPG (Inoue et al. 2015), ARF3 (Kondo et al. 2012), NECP1 (Ritter et al. 2003), and SNX4 (Traer et al. 2007), are involved in endosome formation and trafficking. Others, including NAPG (Stenbeck 1998), SYN2 (Cesca et al. 2010), SVOP (Janz et al. 1998), and EXOC8 (Guo et al. 1999), play key roles in vesicle docking and membrane fusion. Both SYN2 and NAPG have been shown to be disrupted in AD (Nie et al. 2017; Scheff and Price 2003; Sultana et al. 2006). This suggests that normal endo/exocytosis dynamics are affected in aging *Aplysia* SN as well as FL LOAD, possibly contributing to cognitive impairment. Transport of cellular cargo to and from the synapse in response to synaptic activity is also central to synapse function and health (Guillaud et al. 2020; Hafezparast et al. 2003).

Both aging *Aplysia* SN and FL LOAD exhibit downregulation of DCTN6, a component of the dynein/dynactin complex that mediates retrograde transport, and RAB6A, the small GTPase that activates dynein-mediated transport (Yamada et al. 2013). This suggests common impairment of retrograde movement of cellular cargo. Similarly, common downregulation of KIFAP3, a key component of the kinesin motor, suggests that anterograde transport is impaired as well (Yamazaki et al. 1996). Furthermore, previously mentioned STAU2 and ELAVL4 both participate in kinesin-mediated transport of mRNAs from the nucleus to neurites (Bronicki and Jasmin 2013; Tang et al. 2001). Anterograde transport of mitochondria and mRNA via kinesins is crucial for synapse health, learning, and memory, and disruptions of this process are associated with several neurodegenerative disorders (Guillaud et al. 2020). Disruption of mitochondrial transport in neurons also impairs mitochondrial homeostasis, which has been suggested to play a central role in many neurodegenerative disorders (Sheng and Cai 2012).

Mitochondrial dysfunction is a classic hallmark of neural aging and AD (Ferguson et al. 2005; Grimm and Eckert 2017; Ojaimi et al. 1999). Due to the energy-intensive activity of neurons, any disruption in metabolic output can adversely affect signaling and synaptogenesis. The downregulation of several genes in common between *Aplysia* SN aging and FL LOAD suggest similar metabolic impairments. Downregulation of PKFM, the enzyme of the first committed step of glycolysis, but upregulation of glucose sensor and PFKM inducer MondoA, suggests common perturbation of glycolysis homeostasis (Sans et al. 2006). Furthermore, two components of the malate-aspartate shuttle (MAS), GOT2 and MDH1, are commonly downregulated. Disruption of MAS results in decoupling of cytosolic and mitochondrial NAD + /NADH ratios, which has been demonstrated to have adverse effects on mitochondrial metabolism and induce senescence (Bradshaw 2019; Broeks et al. 2019; Lautrup et al. 2019; Xu et al. 2020). Another common downregulated gene, PCCB, is critical for proper functioning of the mitochondrial tricarboxylic acid cycle (TCA) and has also been shown to be downregulated in a mouse model of AD (Franco et al. 2019). Dysfunction of PCC results in altered concentrations of TCA intermediates and accumulation of toxic metabolites, which decreases the activity of pyruvate dehydrogenase (PDH), the beta isoform of which is also downregulated (Wongkittichote et al. 2017). In addition to regulators of glycolysis and the TCA cycle, several components of mitochondrial oxidative phosphorylation are also commonly downregulated. These include components of mitochondrial respiratory complex I (NDUFA10, NDUFV1), cytochrome C (CYCS), which links complexes III and IV, and CISD1, which regulates maximal mitochondrial energy output (Kalpage et al. 2019; Paddock et al. 2007; Wang et al. 2017). These transcriptional signatures suggest similar impairment of mitochondrial energy metabolism in both *Aplysia* SN and FL LOAD. In addition to metabolic impairment, mitochondrial dysfunction also contributes to disrupted Ca^++^ buffering in normal aging and AD (Pandya et al. 2015).

Proper mitochondrial Ca^++^ regulation is critical not only for proper mitochondrial homeostatic functions but also for synaptic signaling (Gleichmann and Mattson 2011; Marchi et al. 2018; Satrustegui et al. 1996). In neurons, mitochondria act as critical sinks and reservoirs for Ca^++^ during signaling events. The signaling pathways that target CREB discussed earlier are themselves dependent upon tightly regulated Ca^++^ signaling (Augustine et al. 2003). Impairment of mitochondrial Ca^++^ homeostasis has been shown to contribute to AD-associated proteinopathies and has even been suggested to be the proximal cause of AD (Calvo-Rodriguez et al. 2020; Jadiya et al. 2019; Tong et al. 2018). Three genes downregulated in both aged *Aplysia* SN and FL LOAD, namely, GDAP1, TUSC2, and GN5B, play an important role in mitochondrial Ca^++^ regulation, suggesting that aged *Aplysia* SN suffer similar disruptions of mitochondrial Ca^++^ dynamics as human FL LOAD (Gonzalez-Sanchez et al. 2019; Kang et al. 2018; Uzhachenko et al. 2014, 2017). Mitochondrial impairment results in energy deprivation, generation of reactive oxygen species (ROS), and elevated Ca++, which contribute to protein aggregation and associated endoplasmic reticulum (ER) stress. Sensors for these stressors converge in a single signaling process known as the integrated stress response (ISR) pathway.

Induction of the ISR results in decreased global translation via phosphorylation of eukaryotic initiation factor 2 (eIF2) and increased transcription of transcription factors in the activating transcription factor family, particularly ATF4 (Costa-Mattioli and Walter 2020; Pakos-Zebrucka et al. 2016). Increased proteostatic stress in AD due to Aβ plaques and tau neurofibrillary tangles has been demonstrated to increase eIF2 phosphorylation, suggesting increased ISR activity in AD (Chang et al. 2002; Ferrer 2002; Hernandez-Ortega et al. 2016; Hoozemans et al. 2005, 2009). Several putative orthologs upregulated in both aged *Aplysia* SN and FL LOAD are stress-induced genes, including DDIT4L (Cuaz-Perolin et al. 2004; Shoshani et al. 2002; Wang et al. 2003), BTG1 (Cho et al. 2003; Yuniati et al. 2019), SSR1 (Nagasawa et al. 2007), ANKZF1 (Tran et al. 2011; van Haaften-Visser et al. 2017), NFIL3 (Tamai et al. 2014), MUC1 (Olou et al. 2020), GAD45G (Liebermann and Hoffman 2008), and BIRC3 (Hamanaka et al. 2009; Warnakulasuriyarachchi et al. 2004). BTG1 enhances ISR signaling via interaction with ATF4 upon activation (Yuniati et al. 2016). Chronic induction of the ISR and resulting changes in the transcriptional and translational landscape of neurons has been suggested to play a role in disruptions of CREB-mediated learning and memory in AD (Hernandez-Ortega et al. 2016). NFIL3 has been shown to specifically inhibit CREB (MacGillavry et al. 2009). Similarly, upregulation of DDIT4L and NEO1 has been demonstrated to result in decreased neurogensis with impaired cognitive outcomes (Chen and Shifman 2019; Di Polo 2015; Metzger et al. 2007; Morquette et al. 2015; Shifman et al. 2009). Activation of the ISR also results in the secretion of cytokines that activate receptors in the toll-like and interleukin-like receptor (TIR) family (Abdel-Nour et al. 2019; Deng et al. 2004; Iwasaki et al. 2014). Activation of these TIR initiates signaling cascades that result in the translocation of transcription factors NF-kB and AP-1 to the nucleus and recruitment of pro-survival and proinflammatory genes.

Increased activation of proinflammatory signaling cascades recruited by the ISR has also been demonstrated to be increased in AD (Colangelo et al. 2002). Positive feedback of this proinflammatory loop has been proposed to induce chronic neuroinflammation and contribute to neurodegenerative consequences in AD (Jones and Kounatidis 2017; Ju Hwang et al. 2019; Lindsay et al. 2021; Uddin et al. 2021). For example, induction of miRNAs by NF-κB in AD directly results in the downregulation of previously discussed SYN2 (Lukiw 2012). Several genes that participate in and are recruited by the signaling cascades downstream of TIR are upregulated in both *Aplysia* SN aging and human FL LOAD (Fig. 2), including MYD88, MAP3K8 (Chorzalska et al. 2017), and MKNK2 (Bao et al. 2017; Xu et al. 2018). Furthermore, NEO1discussed previously exhibits strong proinflammatory effects (Chen and Shifman 2019; Fujita and Yamashita 2017; Shifman et al. 2009). Most significantly, many core components of the quintessential proinflammatory signaling cascade, NF-kB signaling, are commonly upregulated. NF-KB1, also known as p105, is an NF-kB family protein that, upon phosphorylation as a result of MYD88 activation, is degraded by the proteosome. This liberates MAP3K8, which initiates the AP-1 branch of proinflammatory signaling and produces the p50 NF-kB subunit, which is then recruited into homodimers or heterodimers with p65 to activate downstream NF-kB target genes (Beinke et al. 2004). Several of these target genes are commonly upregulated, including NFKBIA (Hay et al. 1999; Sun et al. 1993), BCL3 (Bours et al. 1993; Caamano et al. 1996; Edwards et al. 2015; Saito et al. 2010), and BIRC3 (Hu et al. 2004; James et al. 2006; Simon et al. 2007). Common upregulation of key genes in this pathway suggest that increased proinflammatory signaling as a result of increased cellular stress is a relevant component of *Aplysia* SN aging and FL LOAD. However, few of these relationships have been experimentally validated in *Aplysia*.

While these genes have been observed to play key roles in human neurodegenerative disease, orthologs of these genes have been demonstrated to have conserved function and stress-associated upregulation and function in invertebrate models. Molluscan orthologs of BTG1 (Peng et al. 2014), NFIL3 (Li et al. 2017), MYD88 (Zhang et al. 2015), and BIRC3 (Wang et al. 2016) have been demonstrated to be activated by biotic and abiotic stressors in bivalves. Several other dysregulated orthologs, including NAPG (Clary et al. 1990), SNX4 (Nemec et al. 2017), EXOC8 (Guo et al. 1999), ANKZF1 (Tran et al. 2011), and DDIT4L (Reiling and Hafen 2004) have conserved function between humans and models considered more divergent from humans than *Aplysia* (Moroz et al. 2006), including ecdysozoans like *Drosophila* and *C. elegans* and even yeast. Thus, we believe it plausible that dysregulation of these genes will have similar outcomes in *Aplysia* SN as observed in human neurons.

Differential expression of genes shared between *Aplysia* SN aging and FL LOAD represents critical pathways that are disrupted in aging and neurodegenerative disease, including mitochondrial homeostasis, energy metabolism, vesicle dynamics, cellular cargo transport, Ca^++^ homeostasis, and synaptic plasticity (Di Paolo and Kim 2011; Haas 2019; Jang et al. 2018; Lopez-Otin et al. 2013; Martinez et al. 2017; Wong et al. 2020; Wu et al. 2019; Yin et al. 2016a). Although the hallmark pathologies of AD are only known in humans, these data suggest that, while the proximal source of neuronal stress may be different, similar transcriptional changes as a result of cellular stress underpin cognitive impairment in both *Aplysia* SN aging and AD. Indeed, the commonalities between aging *Aplysia* SN and FL LOAD expression patterns make sense in light of the current understanding that normal brain aging and dementias like AD are parts of a continuum of neurodegenerative outcomes associated with aging (Franceschi et al. 2018). While surface receptors and downstream effectors have diverged and specialized differently over the course of evolution, these data suggest that orthologous signaling cascades and their disruption as a result of age-associated stressors are conserved between the human frontal lobe and *Aplysia* sensory neurons. We strongly believe that these results, in addition to previous studies, demonstrate the excellent applicability of *Aplysia* as a multivalent model for the study of AD and ADRD.

## Supplementary Information

Below is the link to the electronic supplementary material.

Supplementary file1 (XLSX 93 KB)

Supplementary file2 (XLSX 790 KB)

## Data Availability

Data used in this study is freely available from the cited publications and public databases from which it was sourced as described in the text.

## Code Availability

Code used for this study is available at the following GitHub repository: [https://github.com/Nicholas-Kron/Kron_Aplysia_Alzheimer-s_Model].
